# Bacteriology and Surgical Technique for Nurses

**Published:** 1901-09

**Authors:** 


BACTERIOLOGY AND SURGICAL TECHNIQUE FOR NURSES. By Emily M. A. Stoney, Superintendent of the Training School for Nurses, St. Anthony's Hospital, Rock Island, Ill.; author of Practical Points in Nursing, etc. Illustrated. Pp., 190; price, $1.25. Philadelphia: W. B. Saunders & Co. 1900.

The amount of bacteriology necessary for nurses to know is very clearly set forth in this book. Surgical technique is so intimately associated with bacteriology that a knowledge of the former implies a knowledge of the other. Nurses, to appreciate asepsis, must know the why and wherefore. Bacteriology is the basis of successful surgery. This little book has been prepared by one long trained in the teaching and the practice of nursing, and is therefore a valuable work for beginners, as well as those actively engaged in nursing and teaching. T. J. B.

